# Deployable Lab-on-a-Chip Sensor for Colorimetric Measurements

**DOI:** 10.3390/mi14112102

**Published:** 2023-11-16

**Authors:** Stefan Gassmann, Till Schleifer, Helmut Schuette

**Affiliations:** Department of Engineering, Jade University of Applied Sciences, 26389 Wilhelmshaven, Germany

**Keywords:** lab-on-a-chip, microfluidic, colorimetric, environmental monitoring

## Abstract

The infield measurement of nutrients, heavy metals, and other contaminants in water is still a needed tool in environmental sciences. The Lab-on-a-chip approach can develop deployable instruments that use the standardized analytical assay in a miniaturized manner in the field. This paper presents a Lab-on-a-chip platform for colorimetric measurements that can be deployed for nutrient monitoring in open water (oceans, rivers, lakes, etc.). Nitrite was selected as an analyte. Change to other analytes is possible by changing the reagents and the detection wavelength. In this paper, the principle of the sensor, technical realization, setup of the sensor, and test deployment are described. The sensor prototype was deployed at the Jade Bay (German Bight) for 9 h, measuring the nitrite value every 20 min. Reference samples were taken and processed in the lab. The work presented here shows that an infield measurement using a colorimetric assay is possible by applying Lab-on-a-chip principles.

## 1. Introduction

Colorimetric assays for the detection of analytes in liquid samples are well-established methods. The applications range from quality control in drinking water to the monitoring of open water like lakes, rivers, and oceans to the control of process water to monitor substances in body liquids like blood, urine, and saliva [1,2,3,4]. The protocols are standardized, and automation for the lab processes exists. But in most cases, the measurement takes place in the lab. So, the sample needs to be collected, preserved, and processed in the lab. The result of the measurement is given with a certain time delay. A direct measurement at the sample site is often not possible. To overcome this drawback, different approaches are taken. Other techniques for the quantitative measurement of the analyte, like electrical, optical, or biological sensors, have been used [5,6,7]. To use the standardized colorimetric method in the field, the whole lab process needs to be automated. This is carried out, for example, using the nutrient analyzer Micromac-C from Systea (Systea S.p.A., Anagni, Italy) and the Nutrient Analyzer from SubCTech (SubCTech, Kiel, Germany). Most of them are heavy and bulky and cannot be installed in typical water sampling sites. To overcome this problem, miniaturization can be carried out using microfluidic techniques. This is also known as Lab-on-a-chip, where lab procedures are miniaturized. The advantage of this is the usage of the standardized method, which gives comparable results to the lab procedure, the reduction in needed reagents and waste, and long autonomy. A review of microfluidic approaches for water monitoring is given in [8].

The approach to creating an autonomous colorimetric sensor for use in open water using microfluidics is presented here. The selected analyte is nitrite, which is a major parameter for understanding the aquatic system in the coastal environment. Nitrite is a nutrient and an excretion product of phytoplankton and is an important part of the nitrogen cycle. Excessive concentration is dangerous to human health and aquatic organisms [9]. 

The advances in nutrient detection in water over the last five years are shown in the review from Li [10]. The review is structured by analytes and includes a section about nitrite sensors. Li shows the different microfluidic nitrite sensors and recent advances in this field. Unfortunately, the deployment ability is not a major criterion in his review. Deployable microfluidic sensors for nutrients come from a research group at the University of Southampton. Beaton et al. [11] showed a sensor for nitrate and nitrite, which is deployable for several months in a depth down to 6000 m. This sensor used a continuous flow method and a sophisticated method of storing the reagents and waste in bags outside of the pressure-proof housing. Nightingale et al. [12] developed a droplet-based sensor for nitrate and nitrite. This sensor used a very small amount of reagent and was deployed for three weeks in a tidal river. The sensor presented here is one of the few nitrite sensors that is deployable and can work totally autonomously in the field. It uses a continuous flow method compared to the sensor presented in [11]. The advantage of the sensor presented here lies in the light coupling to the absorption cell by optical fibers and in the easy handling of reagents and standards.

In the paper, the principle of the sensor, the technical realization, the setup of the sensor, and the test deployment are described.

## 2. Materials and Methods

The complete sensor design, preparation, and deployment are described in this section.

### 2.1. Sensor Design

**Figure 1:**
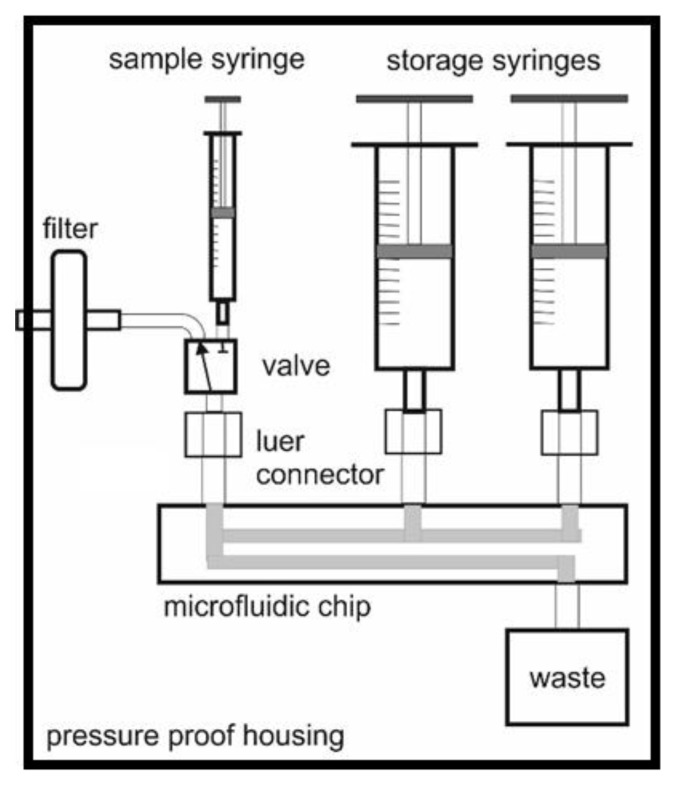
Principle of the sensor design. All elements are inside the pressure vessel. For the reagent and a calibration solution, 20 mL syringes are used. This makes the setup as easy as the setup of an infusion pump.

The sensor should perform all tasks for colorimetric measurement. For the selected analyte, it consists of filtering the sample, mixing the sample with the reagent, waiting for the chemical reaction to complete, and measuring the absorption at the defined wavelength for the reaction product. The main goal of the sensor design is easy handling. Setting up the system should be possible without major microfluidic knowledge. Sources of errors like wrong connections, leaks, or the introduction of large amounts of air should be minimized by design. That is why a syringe-based design was used. The number of parts should be minimized, and easy solutions, e.g., for pumping, should be preferred. The different variants that were discussed during the development process are described in [13]. The final version is described here:

Figure 1 shows the realized principle of the sensor. All liquids are stored inside the pressure vessel. The housing must withstand the hydrostatic pressure according to the deployment depth. Every needed liquid (e.g., reagents, washing, and calibration liquids) has a syringe as a storage container. These could contain 20 mL or a higher volume to have enough liquid available. The pumping of these liquids can be realized using a simple linear actuator. The significant advantage of the usage of syringes is that the setup of the sensor is very easy. It is enough to mount the syringes to the luer adapters. Dead volume and the chance to enter large amounts of air into the system are minimized. The combination syringe and linear actuator ensure easy pumping and well-controlled flow rates inside the chip. A 1 mL syringe is used as a pump for the sample. For this, a 3/2-way valve is connected to the filter. For aspiration of the sample, the valve opens the path to the filter. The sample enters the system with overpressure and can expand in the syringe. To deliver the sample to the chip, the valve is switched, and the sample enters the chip without overpressure. The waste container is located inside the pressure vessel. This is needed to avoid changing backpressure to the fluidic system when the sensor is deployed at different water levels. Because of the needed volume and an easy way to remove the waste from the system, the waste container is a removable item connected to the microfluidic chip. 

The realized sensor consists of the following parts: the microfluidic chip where the reaction and absorption measurement take place; the pump for the sample; the pumps for the reagent, wash, and standard solutions; the waste container; and the housing. All parts are described in the following sections. 

#### 2.1.1. Housing

For the housing, an established and easily available system was selected. The 4″ watertight enclosure from BlueRobotics (www.bluerobotics.com accessed on 20 September 2023) with the acrylic 300 mm tube and two aluminum end caps are used. In one end cap, two cable penetrators were added, one for the sample inlet tube and one for the USB cable. The total outer measurements are about 120 mm in diameter and 340 mm in length.

The inner diameter of the housing is 100 mm. So, all other parts are designed in a way to fit in the 100 mm diameter tube. This is an extra requirement for the microfluidic chip, which must be designed as a circular disc.

#### 2.1.2. Microfluidic Chip

The microfluidic chip is the part where the colorimetric assay takes place. All other parts in the system are there to support the operation of this chip. The microfluidic chip is connected to the fluidic ports of the valve and the syringes by a chip holder. The chip holder is a 10 mm thick PMMA disc that contains the Luer connectors to the syringes and a valve connector. On the other side of the Luer connectors, grooves for O-rings are milled. The sealing is conducted using O-rings measuring 0.8 × 1.25 mm. Four screws press the chip against the holder. Using this approach, the chip can be exchanged easily.

**Figure 2:**
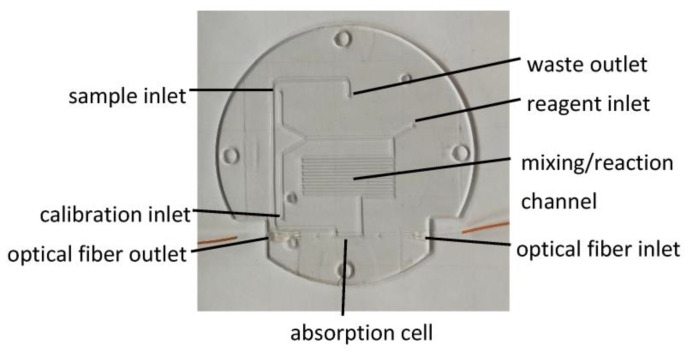
The microfluidic chip. The chip is built from PMMA as a 95 mm diameter disc to fit the housing. A 400 × 400 µm mixing/reaction channel with a length of 45 cm, as well as a 10 mm absorption cell, is created.

The chip itself is a circular disc of 95 mm diameter. It contains the fluidic connections from the sample inlet, the reagent inlet, and the calibration inlet to the mixing/reaction channel, the absorption cell, and the waste outlet (see Figure 2). The fluidic channels have a depth of 400 µm and width of 400 µm. The mixing channel has the same cross-section and a length of 450 mm. In the absorption cell, the optical path has a length of 10 mm. The light source is coupled in by an optical fiber, and on the other end, the light is transported to the photodiode also by an optical fiber (see Figure 2). The procedure to implement is described in [14]. The difference here is that as a light source, an LED of the LV T6SG (OSRAM Opto Semiconductors, Regensburg, Germany) type is used. The coupling to the optical fiber was made using a self-made PCB holder for the FC/PC connector for optical fibers. 

The microfluidic chip is made of PMMA-type PLEXIGLAS XT (Röhm GmbH, Darmstadt, Germany) with a thickness of 2 mm. The channels are created via micro-milling using the Mini-Mill GX (Minitech Machinery, Norcross, GA, USA). After the milling process, the channels are covered with a 0.4 mm thick PMMA sheet type PLEXIGLAS GS (Röhm GmbH). The connection is made via solvent bonding.

#### 2.1.3. Sample Inlet and Pump

**Figure 3:**
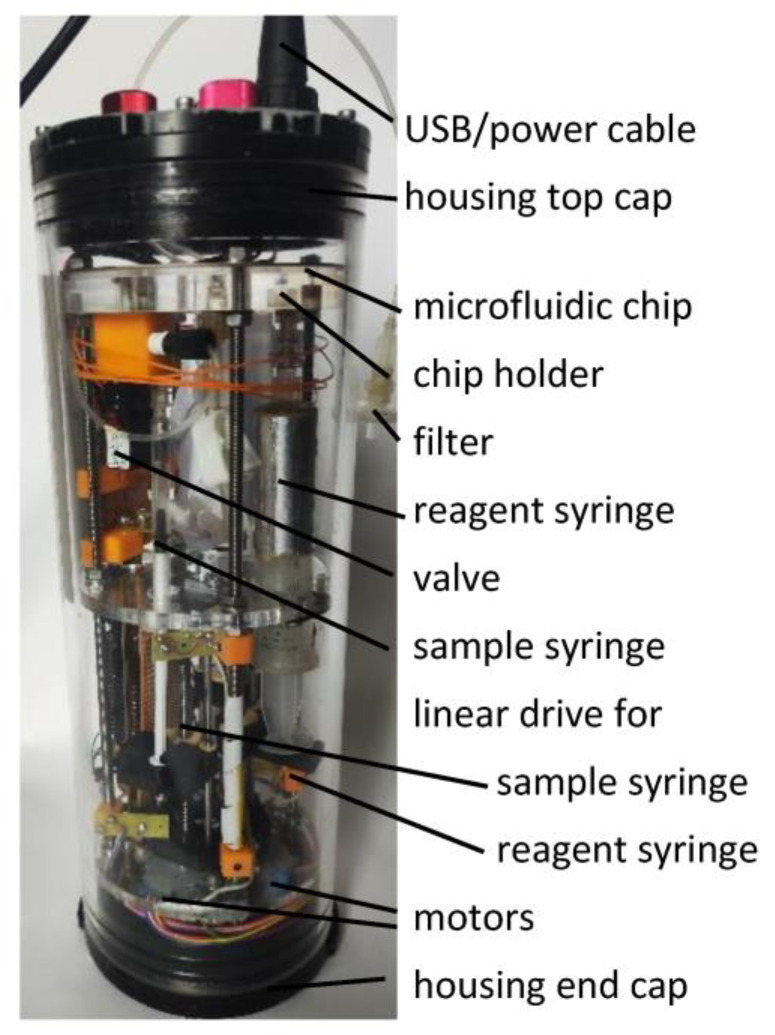
The realized prototype ready for deployment; the elements named in the description are marked. The tube and the end caps are commercially available. All other parts needed to be developed to fit the inner space of the housing.

For the sample inlet, a syringe filter (0.45 µm, PES) is connected to a tube at the outside of the housing for easy replacement. The tube is connected to a 3/2-way valve (6144, Bürkert GmbH & Co KG, Ingelfingen, Germany). The middle port of the valve travels to a 1 mL syringe. This syringe can be either connected to the sample inlet or to the microfluidic chip. The sample can be drawn in by connecting the syringe to the sample inlet using the valve. To deliver the sample to the microfluidic chip, the valve needs to be switched. By pushing the 1 mL syringe plunger with the linear actuator, the sample is delivered to the microfluidic chip. Using this approach, the pressure difference that can result from different deployment depths can be released when the samples are drawn in and kept away from the microfluidic chip. The 1 mL syringe is actuated by a linear actuator consisting of a geared stepper motor (28BYJ-48) and a lead screw dryspin DST-LS-6.35X2.54-R (Igus GmbH, Köln, Germany) (see Figure 3). 

#### 2.1.4. Pumps for Reagent and Other Solutions

The reagent and other liquids, like solutions with known concentrations for automatic calibration, are stored in 20 mL syringes. So, the handling will be easy, and no special knowledge about microfluidic connectors is needed. The syringes are connected by luer connectors to the chip holder. The actuation of the plungers is carried out via a simple linear actuator consisting of a geared stepper motor (28BYJ-48) and a M3 lead screw (see Figure 3). The plunger needs only a forward motion to push the liquid from the syringe to the microfluidic chip, so no clamp for the syringe plunger is needed. 

#### 2.1.5. Waste Storage

The reaction product of the sample and reagent cannot be pumped back to the water source. The waste is stored in the housing of the sensor for later disposal. For this, a bag with a luer connector is used. The luer connector is placed in the middle of the chip holder. So, the waste bag fills the middle area of the housing. For easy waste removal, a clamp is added between the luer connector and the bag.

#### 2.1.6. Electronic Control System

For the control of the system, a Feather M0 Adalogger (Adafruit Industries, LLC, New York, NY, USA) is used. Data storage takes place on the micro-SD card of the Adalogger board. A small display FeatherWing OLED (Adafruit Industries, LLC, New York, NY, USA) helps to show the needed steps during setup and is deactivated for the deployment time. Special stepper motor drivers were developed to fit inside the end cap of the housing to save space. For the control of the LED, the current regulator BCR421U (Infineon AG, Munich, Germany) is used, and the readout of the photodetector PF521 (Roithner Lasertechnik GmbH, Vienna, Austria) is carried out via a circuit for photodiode readout: AS89010 by ams -OSRAM AG (Premstsetten, Austria).

The Adalogger controller communicates via a USB cable to a control PC. The PC is there for starting the measurement cycle, for displaying status information, and for sensor data readout and evaluation. The micro-SD card of the Adalogger is connected as a mass storage device to the PC, so data readout is possible without opening the housing. The PC can be disconnected during the measurements.

The power is also delivered over the USB cable. The system runs only on 5V and can be operated by a power bank. 

### 2.2. Chemical Reaction

Here, the presented sensor has all the modules needed to perform colorimetric measurements. By changing the reagent and the detection wavelength, other analytes can be quantified. The sensor is tailored for measuring Nitrite using the Griess assay. The selected assay can be used for water examination according to the standards EPA 354.1 [15], APHA 4500-NO2-B [16], and DIN EN 26 777 [17]. The chemical reaction used here was first reported by Johann Peter Griess in 1879 and is nowadays a standard method for the quantitative analysis of nitrite. The chemical reaction has two steps. First, nitrite reacts under acidic conditions with sulfanic acid and forms a diazonium cation. Second, this cation couples to the amine 1-naphthylamine to form a red-violet azo dye. The concentration of the azo dye is proportional to the concentration of the nitrite ions and is measured via absorption at a wavelength of 540 nm [18].

### 2.3. Sensor Preparation

The Griess reagent needs to be prepared and stored in the sensor before deployment. Here, the Merck nitrite test 1.14776.0001 [18] is used. The reagent is delivered as a powder. Four micro spoons are dissolved in 20 mL of de-ionized water. The reagent is drawn in the reagent syringe through a 0.45 µm filter. The syringe needs to be protected from light with a cover and is mounted to the sensor. In order to remove the air introduced in the connector, some reagent needs to be pumped through the microfluidic chip. The so-prepared reagent is ready to use and can be used for 1 month. 

### 2.4. Measurement Procedure

The system uses 2 syringes for operation. One 1 mL syringe is there to drive the sample through the system. It is connected to a valve (see Figure 3). The syringe sucks 1 mL of the sample water through a 0.45µm filter and pushes it through the channel with a flow rate of 100 µL/min. Before each measurement, this procedure is repeated three times. Then, 1 mL of sample is drawn using the sample syringe and is pumped through the channel toward the absorption cell with a flow rate of 100 µL/min. A 20 mL syringe contains the reagent. During the measurement, 100 µL of the reagent is dosed in the continuous stream of the sample with a flow rate of 100 µL/min. The mixture is taken along the channel where the mixing and chemical reaction take place. After about 3 min, the reaction product reaches the absorption cell. The reference value (transmission value without the reaction product) is taken at the time when just the sample is in the absorption cell. For the calculation of the concentration, the transmission value is taken when the reaction product is in the absorption cell. Using this approach, an extra absorption cell to measure the reference value is not needed. The calculation of the concentration is conducted using the following formula:

c = log (reference value/transmission value) × calibration factor(1)

### 2.5. Calibration Measurement

Calibration measurements were made by preparing 3 different concentrations of Nitrite ions in de-ionized water (0.5 mg/L; 0.25 mg/L; and 0.125 mg/L). The solutions were prepared from the standard solution 1000 mg/L (1.19899, Merck, Darmstadt, Germany).

**Figure 4:**
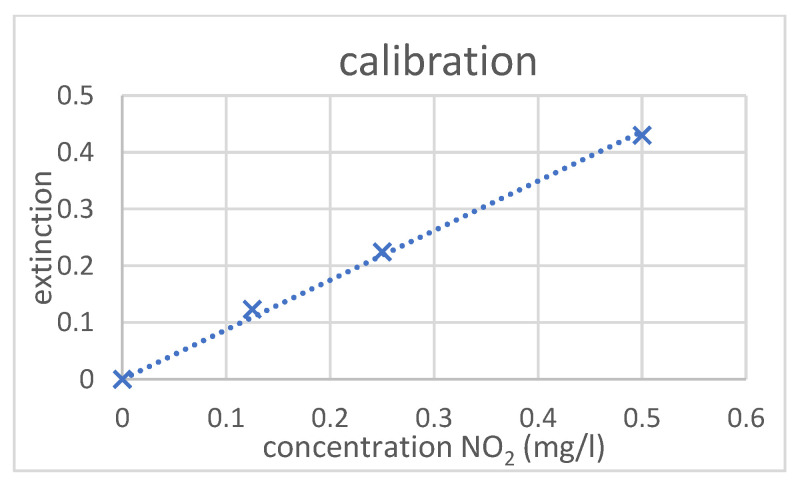
Graph of the calibration measurement.

Figure 4 shows the graph of the calibration measurement. From the calibration curve, the calibration factor is taken via linear regression.

### 2.6. Deployment

**Figure 5:**
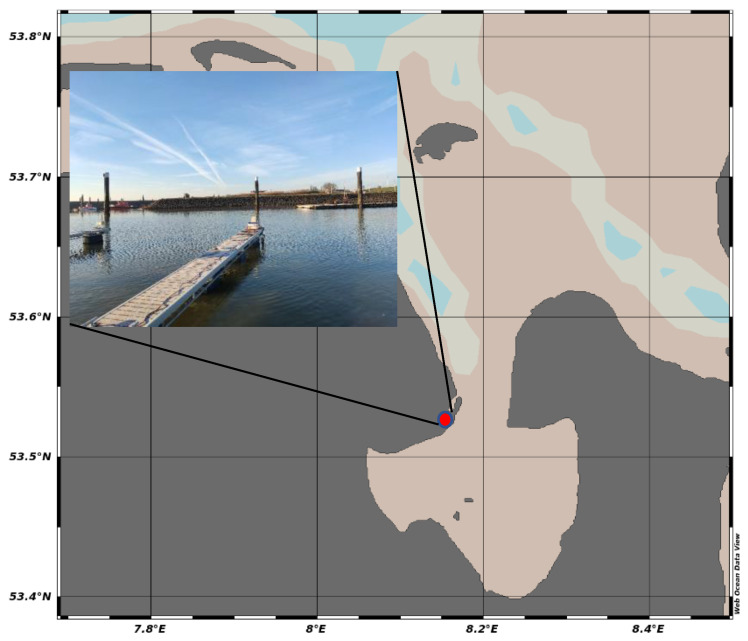
Location and photo of the deployment site.

The deployment test described here took place on 8 February 2023 at the Jade Bay (German Bight). As a deployment site, the Nassau harbor near the inlet of the Jade Bay was chosen (N 53.5147548; E 8.1510548). Figure 5 shows the position and a photograph of the de-ployment location.

The deployment took place from 9 a.m. to 6 p.m. with a measurement frequency of 20 min. The tides at the nearby tide station (Wilhelmshaven, Alter Vorhafen) were low tide at 5:41 a.m. (0.7 m), high tide at 11:17 a.m. (4.1 m), and low tide at 5:38 p.m. (0.6 m). With the time series, the time from the high tide to the low tide is covered. (All times are local times). 

### 2.7. Reference Measurements

At the time interval of two hours, reference samples were taken. For this, 50 mL falcon tubes were filled with water and stored in a cool place until measurement in the evening of the same day. The Nitrite measurement of the reference samples was conducted according to the Nitrite protocol described in [19,20]. The Griess reagent was prepared by mixing the NEDD solution (0.2%, made from 100 mg N-1-naphthylethylenediamine dihydrochloride (NEDD, p.a., Merck) dissolved in 100 mL ultrapure water) and Sulfanilamide solution (2%, made from 2 g Sulfanilamide (p.a., Merck) dissolved in 100 mL 10% (*v*/*v*) HCl) in the ratio 1:1. Thirty µL of this reagent was added to a 300 µL sample or calibration to a 96-well plate (flat bottom-type, polystyrene microtiter plates, Greiner). An incubation time of 30 min at 45 °C was applied. The absorption measurement was performed in a microtiter plate reader Multiscan GO (Thermo-Fischer Scientific, Darmstadt, Germany). 

## 3. Results

The results of the deployment are presented in this Section.

**Figure 6:**
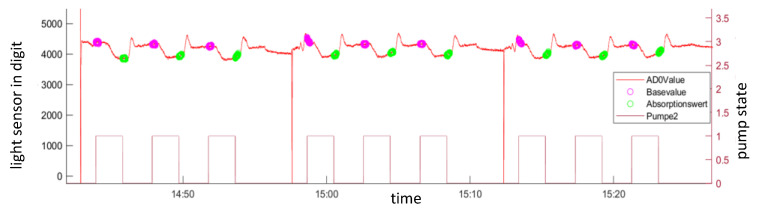
Raw data of the measurements. The transmission value of the absorption cell is shown as AD0Value (red), and the rectangular-shaped curve shows the activity of the reagent pump. Circles show the values that are taken to calculate the concentration (pink = reference value; green = transmission value).

Figure 6 shows the raw data of the light measurement in the absorption cell. It is shown that three measurements are conducted for each sample drawn. This measurement is repeated three times. After these three measurements, a break of 20 min is added. This measurement scheme is freely programmable. 

The high swing of the transmission signal right before the beginning of the measurement is related to a measurement of the background light. The liquid passing through the absorption cell first contains just the sample. At this time, the reference value (see Formula (1)) is taken. In Figure 6, this is marked by pink circles. Then, the reagent pump is switched on, and after the reaction takes place, the colored reaction product reaches the absorption cell. The light sensor signal travels down until it reaches a stable point. Then, the reagent pump is switched off, and the transmission value (see Formula (1)) is taken (see Figure 6 green circles). With these two values, the concentration can be calculated. The measurement in Figure 6 shows a typical artifact for this kind of measurements. After the reagent pump is switched and the liquid without reagent is transported through the absorption cell, the light intensity rises. This happens when the liquids have different refractive indexes. Due to the parabolic shape of the front of the new liquid, a lens effect takes place. 

**Figure 7:**
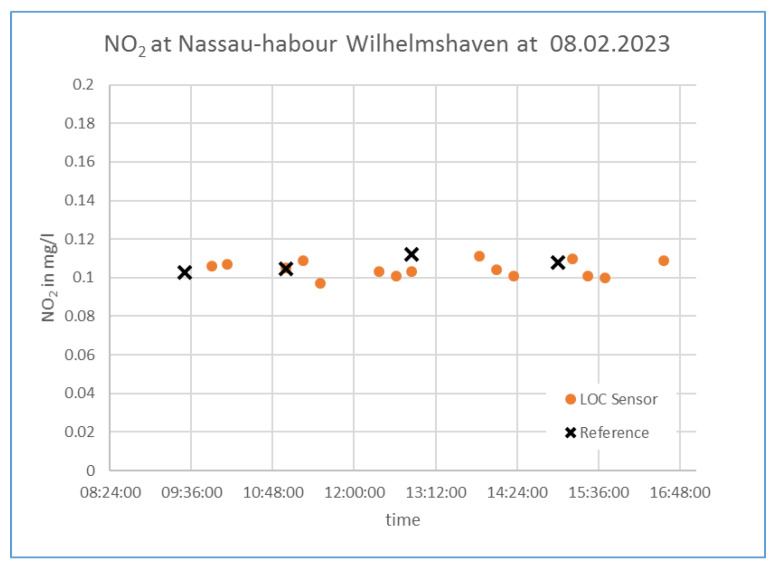
Time series of the concentration measurement of NO_2_ on 8 February 2023 at the deployment site. Dots are the measurement values from the described Lab-on-a-chip sensor. Crosses are the reference measurements.

Figure 7 shows the time series of the deployment of the microfluidic sensor at the Nassau harbor, Wilhelmshaven. Each dot represents the mean value of the three measurements taken of one sample. The crosses show the reference measurements performed at the lab with the above-described procedure. The values show good agreement. 

It was expected to see a difference with the different tides. This difference is only visible in the reference values, where the Nitrite value is about 0.01 mg/L higher near high tide compared to the value at low tide. This tendency is not visible in the measurement values of the presented sensor. The reason for this needs to be investigated. It is possible that the sampling and flushing strategies and/or the absorption cell need to be improved. 

## 4. Conclusions

The presented sensor shows that a colorimetric measurement principle can be miniaturized and taken to the field for a nutrient measurement in seawater. The presented example is for nitrite but can be applied to other analytes as well. An autonomous nutrient measurement system becomes possible using the here-presented lab-on-a-chip approach. 

Nevertheless, there are things to improve in the presented system. The measurement range can be broadened by adding absorption cells with different lengths. The reagent syringe can be made bigger to achieve longer deployment time. Wireless communication, as well as an on-board data evaluation, are features that make this system easier to use.

## Data Availability

Data are contained within the article.

## References

[1] Lindon J.C., Tranter G.E., Koppenaal D.W. (2017). Encyclopedia of Spectroscopy and Spectrometry.

[2] Gummadi S., Kommoju M. (2019). Colorimetric Approaches to Drug Analysis and Applications—A Review. Am. J. Pharmtech Res..

[3] Liu B., Zhuang J., Wei G. (2020). Recent advances in the design of colorimetric sensors for environmental monitoring. Environ. Sci. Nano.

[4] Fernandes G.M., Silva W.R., Barreto D.N., Lamarca R.S., Gomes P.C.F.L., Petruci J.F.D.S., Batista A.D. (2020). Novel approaches for colorimetric measurements in analytical chemistry—A review. Anal. Chim. Acta.

[5] Huang C.-W., Lin C., Nguyen M.K., Hussain A., Bui X.-T., Ngo H.H. (2023). A review of biosensor for environmental monitoring: Principle, application, and corresponding achievement of sustainable development goals. Bioengineered.

[6] Butt M.A., Voronkov G.S., Grakhova E.P., Kutluyarov R.V., Kazanskiy N.L., Khonina S.N. (2022). Environmental Monitoring: A Comprehensive Review on Optical Waveguide and Fiber-Based Sensors. Biosensors.

[7] Baranwal J., Barse B., Gatto G., Broncova G., Kumar A. (2022). Electrochemical Sensors and Their Applications: A Review. Chemosensors.

[8] Saez J., Catalan-Carrio R., Owens R.M., Basabe-Desmonts L., Benito-Lopez F. (2021). Microfluidics and materials for smart water monitoring: A review. Anal. Chim. Acta.

[9] Dutt J., Davis J. (2002). Current strategies in nitrite detection and their application to field analysis. J. Environ. Monit..

[10] Li Z., Liu H., Wang D., Zhang M., Yang Y., Ren T.-L. (2023). Recent advances in microfluidic sensors for nutrients detection in water. TrAC Trends Anal. Chem..

[11] Beaton A.D., Cardwell C.L., Thomas R.S., Sieben V.J., Legiret F.-E., Waugh E.M., Statham P.J., Mowlem M.C., Morgan H. (2012). Lab-on-chip measurement of nitrate and nitrite for in situ analysis of natural waters. Environ. Sci. Technol..

[12] Nightingale A.M., Hassan S.-U., Warren B.M., Makris K., Evans G.W.H., Papadopoulou E., Coleman S., Niu X. (2019). A droplet microfluidic-based sensor for simultaneous in situ monitoring of nitrate and nitrite in natural waters. Environ. Sci. Technol..

[13] Gassmann S., Schütte H., Thoma C. Macro-Micro-Interface for a Microfluidic Nitrite Sensor. Proceedings of the ACTUATOR18 International Conference on New Actuators and Drive Systems.

[14] Gassmann S., Schuette H., Thoma C. Colorimetric microfluidic Nitrite sensor with optical fiber coupling. Proceedings of the IECON 2016—42nd Annual Conference of the IEEE Industrial Electronics Society.

[15] Methods for the Chemical Analysis of Water and Wastes (MCAWW). https://www.nemi.gov/methods/method_summary/5777/.

[16] (1992). Standard Methods For the Examination of Water and Wastewater.

[17] (1993). Water Quality—Determination of Nitrite—Molecular Absorption Spectrometric Method.

[18] Merck. Spectroquant Nitrite Test Nr. 1.14776.0001, 2021, Data Sheet, File Name: 114776e.pdf. https://www.emdmillipore.com/US/en/product/Nitrite-Test,MDA_CHEM-114776.

[19] Schnetger B., Lehners C. (2014). Determination of nitrate plus nitrite in small volume marine water samples using vanadium(III)chloride as a reduction agent. Mar. Chem..

[20] Miranda K.M., Espey M.G., Wink D.A. (2001). A Rapid, Simple Spectrophotometric Method for Simultaneous Detection of Nitrate and Nitrite. Nitric Oxide.

